# Green Chemistry Applications Using Complexing Materials for Water Treatment

**DOI:** 10.3390/polym17111467

**Published:** 2025-05-25

**Authors:** Nicoleta Mirela Marin

**Affiliations:** 1 National Research and Development Institute for Industrial Ecology ECOIND, Street Podu Dambovitei No. 57-73, District 6, 060652 Bucharest, Romania; nicoleta.marin@incdecoind.ro; 2 Department of Oxide Materials Science and Engineering, National University of Science and Technology Politehnica Bucharest, 1–7 Gh. Polizu, 060042 Bucharest, Romania; 3 Department of Analytical and Physical Chemistry, University of Bucharest, 4-12 Regina Elisabeta Bd., 030018 Bucharest, Romania

**Keywords:** cellulosic materials, complexing process, adsorption, metal ions, reutilization, wastewater, environmental sustainability

## Abstract

In this study, two complexing materials were obtained for Mn^2+^, Zn^2+^, Fe^3+^ and Cr^3+^ removal from aqueous media. Synthetic cellulose powder (CELL) and cellulosic green material obtained from shredded maize stalk (MS) were modified with direct red 23 (DR 23), a complexing agent, obtaining MS-DR 23 and CELL-DR 23 using batch mode technique. Experimental parameters like interaction time, pH of aqueous solution, and initial concentration of DR 23 were studied to optimize the complexing process. The time necessary to reach equilibrium was 75 min for both cellulose materials. Also, pH 2 was the optimum adsorption value for adsorption of DR 23. The adsorption capacity for MS (56.8 mg/g) was more significant than for CELL (42 mg/g). The applicability of complexing materials was based on checking for Mn^2+^, Zn^2+^, Fe^3+^ and Cr^3+^ (M^x+^) removal. The concentration of M^x+^ retained on cellulosic materials was detected by the atomic adsorption spectrometry method (AAS). The complex formation between DR 23 and M^x+^ was evaluated at pH 2.0, 4.0, 6.0, 8.0 and 10.0. Batch adsorption experiments were conducted to assess the adsorption of M^x+^ onto MS-DR 23 and CELL-DR 23. A high level of M^x+^ adsorption was reported at 4 mg/L. Reusability experiments were conducted and complexing cellulose was used for multiple cycles of M^x+^ removal from aqueous media. Also, the developed complexing materials tested M^x+^ removal from tannery wastewater. Based on experimental study, two complexing materials for metal removal were produced. The good adsorption and regeneration of complexing materials provide an excellent adsorbent for water purification.

## 1. Introduction

Water pollution is a significant issue that generates extensive global discussion [1,2]. In response, green technologies offer promising solutions for enhancing wastewater treatment [3,4,5,6]. These innovative approaches not only tackle the challenges associated with removing inorganic contaminants but also contribute to a more sustainable future [7,8,9]. One particularly effective method is adsorption through complexation interactions, which provides a straightforward and efficient way to eliminate hazardous metals [10,11,12]. By embracing these technologies, we can substantially improve water quality and safeguard the environment [13]. Traditional wastewater treatment methods are often quite expensive and tend to generate additional waste byproducts [14]. These methods typically require significant financial resources for infrastructure, operation, and maintenance. Moreover, the processes can result in leftover sludge and other materials that need further disposal or treatment, adding to the overall environmental impact and costs involved [15]. As a result, there is increasing interest in discovering more sustainable and cost-effective solutions [16,17]. Natural materials and those derived from waste are increasingly evaluated for their abundance, bioavailability, and environmental benefits [18]. Cellulose, a natural polymer existing in nature, shows potential for use in wastewater treatment for pollutant removal [13,19,20,21]. The main functional groups of cellulose are hydroxyl (existing in the C2, C3 and C6 positions in each linear structure of glucose residue) and carbonyl; they enhance the adsorption of heavy metals and organic pollutants through mechanisms like surface complexation, ion exchange, and physical adsorption [22,23,24]. This property makes cellulose materials effective adsorbents for contaminants in wastewater [25,26,27].

Cellulose together with its modified structures was created to obtain a new category of adsorbents with improved properties for retaining metal ions [5,28,29,30,31,32,33]. At the same time, more laboratory investigations are necessary for the modification and isolation of cellulose [34,35,36]. Also, various chemicals are required to extract cellulose, making the process expensive and often environmentally unsustainable [37,38,39]. Despite the great importance of the adsorption capacity of the adsorbent material, the economy of the regeneration step of the process is much less studied than adsorption studies [40].

Until this study, the potential of complexing celluloses MS-DR 23 and CELL-DR 23 had not been employed for Mn^2+^, Zn^2+^, Fe^3+^ and Cr^3+^ removal to the author’s knowledge. As a consequence, the main objective of this study was to obtain new chelating cellulose using DR 23 as chelating agent, cellulose powder (CELL), and cellulosic material derived from shredded maize stalks (MS) for Mn^2+^, Zn^2+^, Fe^3+^ and Cr^3+^ removal. In the experimental part, pH influence, kinetic and equilibrium studies were employed for obtaining new complexing cellulose materials. Also, the stability of the MS-DR 23 and CELL-DR 23 was verified in the presence of the desorption agents in acidic and basic medium. The UV-Vis method was used to estimate the concentration of the complexing agent retained onto cellulosic materials. Also, the adsorption performances of the resulting materials were tested for adsorption of divalent metal ions (Zn^2+^ and Mn^2+^) and trivalent metal ions (Cr^3+^ and Fe^3+^) from polluted aqueous matrices via the AAS method. Metal interaction at different pH values and masses of complexing celluloses, as well as kinetics and equilibrium studies, were evaluated. Regeneration and reuse of complexing CELL-DR 23 and MS-DR 23 in five metal adsorption/desorption cycles were completed. Also, MS-DR 23 and CELL-DR 23 for depolluting real wastewater were tested.

## 2. Materials and Methods

### 2.1. Chemicals

Cellulose powder (Supelco), direct red 23–([[6-[(4-acetamidophenyl)diazenyl]-5-hydroxy-7-sulfonatonaphthalen-2-yl] cabamoylamino]-4-hydroxy-3-phenyldiazenylnaphthalene-2-sulfonate) 30% dye content (Sigma Aldrich, St. Louis, MI, USA), certified reference materials (CRM) mono-element solutions of 1000 mg/L Zn(NO_3_)_2_, Mn(NO_3_)_2_, Fe(NO_3_)_3_ and Cr(NO_3_)_3_, 37% HCl and 50% NaOH from Merck were used in experimental study.

### 2.2. Equipment

For UV-VIS spectrometric analysis, a DR/5000 TM spectrometer (Hach Lange, Berlin, Germany) was utilized. For the quantitative determination of metal ions, a Perkin Elmer PinAcle 900T atomic absorption spectrometer (Perkin Elmer, Norwalk, CT, USA) was employed. The pH of the supernatant and buffer solutions was checked using a HI 255 pH-meter (Hanna Instruments, Nijverheidslaan, Belgium). For the preparation of the complexing celluloses, as well as for the adsorption of metals onto the complexing celluloses, a GFL 3017 horizontal mechanical shaker (Bremen, Germany) was used.

### 2.3. Preparation of Stock Solutions

The stock solution 1 g/L was obtained by weighing an appropriate amount of DR 23, dissolving it in water and subsequently transferring to a 500 mL volumetric flask. Aqueous solutions of different HCl concentrations were prepared by diluting 37% HCl solution for the activation and purification of cellulose materials. Simultaneously, phosphate, acetate and carbonate buffer solutions were prepared. The buffer solutions employed were: phosphate buffer for pH = 2.0, acetate buffer for pH = 4.2, acetate buffer for pH = 6.0, phosphate buffer for pH = 8.0 and carbonate buffer for pH = 10.2, respectively.

### 2.4. Procedure for Purification and Activation of Cellulosic Materials

Initially, the powdered cellulose (≈4 g) was washed and hydrated and subsequently transferred to a glass column over which 30 mL of 4 M HCl was passed with a flow rate of 0.4 mL/min. After activation and purification, the cellulose was washed with demineralized water until the reaction for chloride ion was negative (verification with 0.02 M Hg_2_(NO_3_)_2_ solution). The cellulose was removed from the column, filtered and left to dry for 48 h at room temperature. Acid treatment of shredded maize stalk was done according to methodologies developed in a previous study [41].

### 2.5. Procedure for the Complexation of Cellulosic Materials in Function of pH

Over 0.05 g MS and CELL, 0.02 L of 315 mg/L DR 23 solution were added. The pH of the studied solutions was pH = 2.0, 4.2, 6.0, 8.0 and 10.2. The pH = 7 was obtained when DR 23 was dissolved in ultrapure water. The mixtures obtained were stirred at 175 rpm, 75 min at T = 25 ± 2 °C. At the end of the stirring, the mixtures were filtered and the amount of DR 23 that was not retained on the cellulose mass was determined spectrometrically. All experimental data were done in duplicate, and the average data were used to determine adsorption capacity (*Q_e_*). The adsorption capacity *Q_e_* (mg/g), obtained at equilibrium for DR 23 adsorption on MS and CELL, was determined by applying the Equation (1):

(1) $$Q_{e}=\frac{\left(C_{i}-C_{e}\right)\cdot V}{m}$$

where *C_i_* and *C_e_* (mg/L) represent the initial concentration of DR 23 and concentration at equilibrium (*e*), m(g) is the mass of dry MS and CELL, and *V*(L) is the volume of DR 23.

### 2.6. Procedure for the Adsorption of Complexing Agent in Function of Contact Time on Cellulosic Materials

Thus, 0.05 g of MS and CELL were weighed into Erlenmeyer flasks. Then, 0.02 L of 315 mg/L DR 23 solution pH = 2 was added to the cellulosic materials. The mixtures obtained were stirred at 175 rpm (25 ± 2 °C) at the following interaction times: 15, 30, 45, 60, 75, and 90 min. After stirring, the celluloses loaded with DR 23 were filtered on filter paper, and the amount of DR 23 retained in the resin mass was determined from the filtered solutions using the calibration curve obtained at the linearity of the spectrometric method. All experimental data were done in duplicate, and the average data were used to determine *Q_t_*.

The adsorption capacity evaluated in function of the interaction time (*Q_t_* (mg/g)) was calculated with the Equation (2):

(2) $$Q_{t}=\frac{\left(C_{i}-C_{t}\right)\cdot V}{m}$$

where *C_t_* (mg/L) represents the concentration of DR 23 at time *t* in the solution.

The percentage of metals removed (R (%)) from aqueous matrices onto MS-DR 23 and CELL-DR 23 were determined by applying Equation (3):

(3) $$R(\%)=\frac{\left(C_{i}-C_{e}\right)}{C_{i}}\cdot 100$$

where *C_i_* (mg/L) are the initial concentration of metals and *C_e_* (mg/L) is the metal concentration at equilibrium.

### 2.7. Procedure Used to Obtain Complexing Celluloses in Function of Initial Concentration of DR 23

Over 0.05 g of cellulosic material, fixed volumes of 0.02 L DR 23 of 105, 210, 315 and 420 mg/L were added in 250 mL Erlenmeyer flasks. The obtained mixtures were stirred for 75 min at 175 rpm and at T = 25 ± 2 °C. At the end of stirring time, the celluloses in complexed form with DR 23 were filtered, and the amount of DR 23 that was not retained in the mass of material was determined spectrometrically based on the calibration curve presented in the experimental part.

### 2.8. Procedure for Evaluating the Stability of Complexing Celluloses MS-DR 23 and CELL-DR 23

For the stability evaluation, samples of 0.5 g each loaded with 39.9 mg/g CELL-DR 23 and 52.4 mg/g MS -DR 23 were used in this regard. Subsequently, 30 mL 2 M HCl and NaOH solution were added to the weighed samples. The resulting mixture were stirred in Erlenmeyer flasks for 30 min at 175 rpm (T = 25 °C). After 30 min of stirring, the samples were kept in standby for 5 min and subsequently filtered. The filtered solutions were analyzed spectrometrically in the 200–800 nm range.

### 2.9. Experimental Procedure for the Adsorption of Mn^2+^, Zn^2+^, Fe^3+^ and Cr^3+^ Using MS-DR 23 and CELL-DR 23 as a Function of pH Solutions

For the removal of Mn^2+^, Zn^2+^, Cr^3+^ and Fe^3+^ on MS-DR 23 and DR 23-CELL, respectively, ≈ 0.05 g of MS-DR 23 (56 mg DR 23/g) and CELL-DR 23 (41 mg DR 23/g) were stirred with 0.03 L solutions pH = 2.0; 6.0; 8.0; 10.2, in which the concentration of Mn^2+^, Zn^2+^, Fe^3+^ and Cr^3+^ was 2.5 mg/L. The mixtures were stirred for 90 min at 175 rpm (T = 25 ± 2 °C). After stirring, the mixtures were filtered and the metal concentration was determined by AAS method.

### 2.10. Procedure for Evaluating Contact Time for Metal Ions Adsorption

To study the time required to reach adsorption equilibrium for the removal of Mn^2+^, Zn^2+^, Cr^3+^ and Fe^3+^ on MS-DR 23 and DR 23-CELL, over 0.5 g of complexing material, 0.05 L solution pH = 10 with a concentration of 4 mg/L was added. The mixtures thus obtained were stirred at 175 rpm (T = 25 ± 2 °C) using the batch method at different time intervals of 15, 30, 45, 60, 75 and 90 min. The equilibrium was considered reached at the time when the concentration of Mn^2+^, Zn^2+^, Cr^3+^ and Fe^3+^ in the measured samples remained constant.

### 2.11. Procedure for Metal Ions Retained on MS -DR 23 and CELL-DR 23

For this purpose, 0.05 g sample of CELL-DR 23 and MS-DR 23 was subjected to mechanical stirring for 90 min at 175 rpm (T = 25 ± 2 °C) with 0.03 L pH = 10 containing 0.5; 1.0; 1.5; 2.0; 2.5; 3.0 and 4 mg/L Mn^2+^, Zn^2+^, Fe^3+^ and Cr^3+^. At the end of stirring time, the concentration of unretained Mn^2+^, Zn^2+^, Cr^3+^ and Fe^3+^ in the mass of the MS-DR 23 and CELL-DR 23 was determined using AAS method. Data obtained at equilibrium were modulated applying Equation (1) and Langmuir Equation (4).

(4) $$\frac{C_{e}}{Q_{e}}=\frac{1}{bQ_{\max}}+\frac{C_{e}}{Q_{\max}}$$

where *Q_max_*(mg/g), *b*(L/mg) are Langmuir constants correlated with the adsorption capacity and affinity of complex materials.

### 2.12. Procedure for Desorption of Metal Ions from Complexing Materials

The complexing materials loaded with 1.11 mg/g Mn^2+^, 0.81 mg/g Cr^3+^, 0.87 mg/g Zn^2+^, and 1.16 mg/g Fe^3+^ on CELL-DR 23 and 1.17 mg/g Mn^2+^, 1.09 mg/g Cr^3+^, 1.07 mg/g Zn^2+^ and 1.18 mg/g Fe^3+^ on MS-DR 23 ≈0.05 g were subjected to desorption with 30 mL of 0.25, 0.5 and 1 M HCl. The obtained solutions were stirred in Erlenmeyer flasks for 30 min in batch mode at 175 rpm (T = 25 ± 2 °C). After 30 min of stirring, the samples were left to stand for 5 min and subsequently filtered, and the filtered solutions were analyzed by AAS.

### 2.13. Procedure for Reuse of Complexing Materials in Several Adsorption–Desorption Cycles

The complexing materials ≈ 0.05 g CELL-DR 23 and MS-DR 23 were stirred with 0.05 L mixed solution of 2 mg/L metal ions at pH = 10 for 90 min at 175 rpm (T = 25 ± 2 °C). After 90 min of stirring, the mixtures were centrifuged and supernatant solution was collected and subjected to AAS analysis to determine the concentration of unretained metal ions on the mass of complexing material. Simultaneously, the solid phases loaded with metal ions were subjected to regeneration. After each regeneration step, the solid phases were reused for five adsorption/desorption cycles.

### 2.14. Procedure for Using Complexing Materials in Wastewater Depollution Processes

For the application of complexing materials in wastewater depollution processes, 0.05 g of material loaded with 39.9 mg/g CELL-DR 23 and 52.4 mg/g MS-DR 23 were used. The obtained mixtures were stirred for 90 min at 175 rpm (T = 25 ± 2 °C) with 0.05 L wastewater matrix. Herein, wastewater matrix was obtained by mixing 0.025 L tannery wastewater with 0.025 L buffer solution pH = 10. The initial concentration (*C_i_*, mg/L) of Mn^2+^, Zn^2+^, Cr^3+^and Fe^3+^ before adsorption on complexing materials was determined and the values obtained were as follows: 1.9 ± 1.75 mg/L Cr^3+^, 1.2 ± 1.06 Fe^3+^, 0.7 ± 0.55 mg/L Mn^2+^ and 0.35 ± 0.22 mg/L Zn^2+^, respectively. At the end of the experiment, the mixtures were filtered and the concentration of metal ions that was not retained on the complexing materials was determined by AAS.

## 3. Results and Discussion

### 3.1. Linearity Study of Analytical Methods Used for the Quantitative Determination of the Complexing Agent DR 23 and for Mn^2+^, Zn^2+^, Cr^3+^and Fe^3+^

The spectrometric methods described below were used to estimate the concentration of the complexing agent and metal ions in the studies aimed at developing the experimental model.

**Figure 1:**
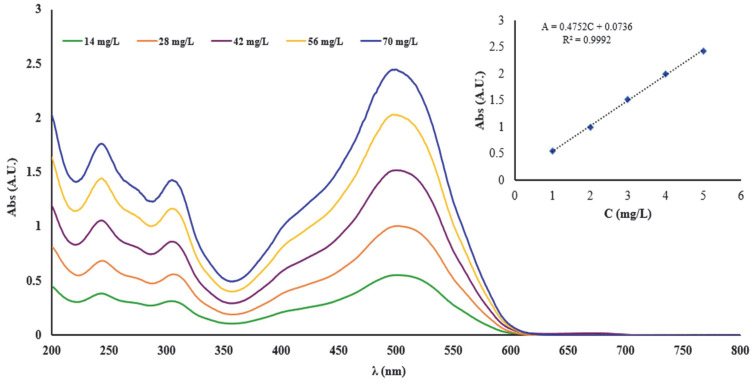
UV-Vis spectra of DR 23 recorded in the range 200–800 nm and calibration curve derived from the UV-Vis spectra read at 504 nm.

Thus, the linearity of the UV-Vis spectrometric method was studied to determine the concentration of the complexing agent used for complexation of the cellulose materials. In this regard, the calibration curve was examined in concentration ranges that varied between 14–70 mg/L DR 23. From the graphical representation of the absorbance (A) read at the 504 nm wavelength (λ) in function of the concentration (C), the calibration line A = 0.4752C + 0.0736 was obtained, and the value of the correlation coefficient (R^2^) was 0.9992. The high value of R^2^ suggests a good linearity of the UV-Vis method over the entire concentration range studied, indicating that Beer’s law was verified (Figure 1).

**Figure 2:**
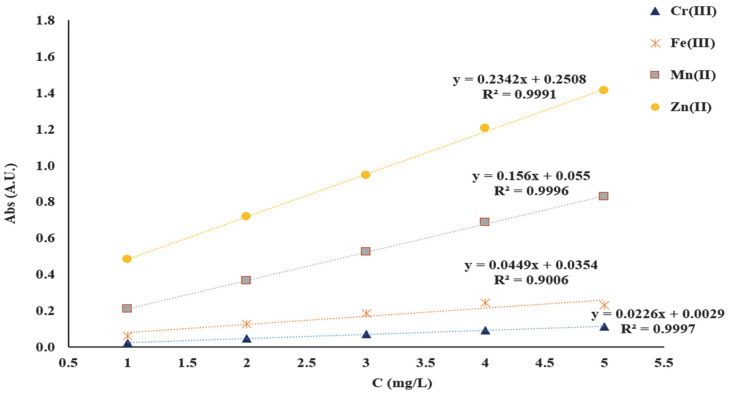
Calibration curves of Cr^3+^, Fe^3+^, Mn^2+^ and Zn^2+^.

The linearity of the AAS method was verified in the range 1–5 mg/L. The solutions for establishing the linearity of the AAS method were prepared from 1000 mg/L CRM stock solution of Zn(NO_3_)_2_, Mn(NO_3_)_2_, Fe(NO_3_)_3_, and Cr(NO_3_)_3_. Initially, working solutions were obtained and these were prepared as follows: 5 mL from each CRM solution of 1000 mg/L was quantitatively transferred to a 50 mL volumetric flask. Subsequently, the solutions for plotting the calibration curves were obtained by diluting the stock solution to achieve 1, 2, 3, 4, and 5 mg/L of Mn^2+^, Zn^2+^, Cr^3+^ and Fe^3+^ necessary for the linearity study of the AAS method. The wavelengths used for the detection of the studied metal ions were as follows: 279.48 nm for Mn^2+^, 213.86 nm for Zn^2+^, 357.87 nm for Cr^3+^ and 248.33 nm for Fe^3+^. To obtain the calibration curves, each standard solution corresponding to each metal ion was analyzed. The calibration curves were obtained, representing the absorbance intensity vs. the concentration in mg/L (Figure 2).

### 3.2. Adsorption Experiments of Complexing Agent DR 23

In order to obtain new complexing materials (MS-DR 23 and CELL-DR 23), experimental parameters such as the influence of the pH in aqueous medium, the contact time between the two phases and the initial concentration of the complexing agent (DR 23) can significantly impact the retention of DR 23 on the two celluloses studied. Thus, in order to evaluate the adsorptive potential of cellulosic materials, laboratory studies were conducted under batch conditions using aqueous systems containing the complexing agent.

Mechanism Proposed for Obtained Complexing Materials

**Figure 3:**
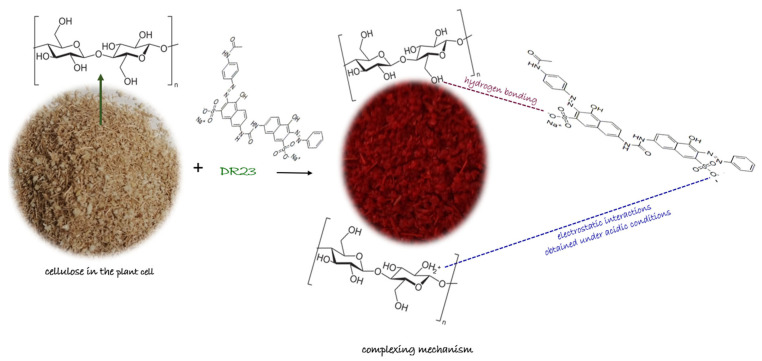
Mechanism proposal for adsorption of DR 23 into cellulosic material.

The proposed mechanism according to the literature data [26,42] for the adsorption of the complexing agent DR23 on the cellulose existing in the structure of the shredded maize stalk (MS) and on the synthetic cellulose (CELL) are presented in Figure 3 and can be described as follows:

(i) Hydrogen bonds may form between the strong hydrogen donor of DR 23 and the hydrogen acceptor of the hydroxyl group in the cellulose structure;
(ii) At acidic pH values, the hydroxyl groups became protonated and act as positively charged species fixing the negative molecules of complexing agent DR 23 through an ion exchange equilibrium. Electrostatic interactions may occur when the negatively charged sulfate (–SO_3_^−^) groups interact with the –OH_2_^+^ group in the cellulose material structure in a strongly acidic environment.

### 3.3. Study of Complexation Cellulosic Materials in Function of pH

**Figure 4:**
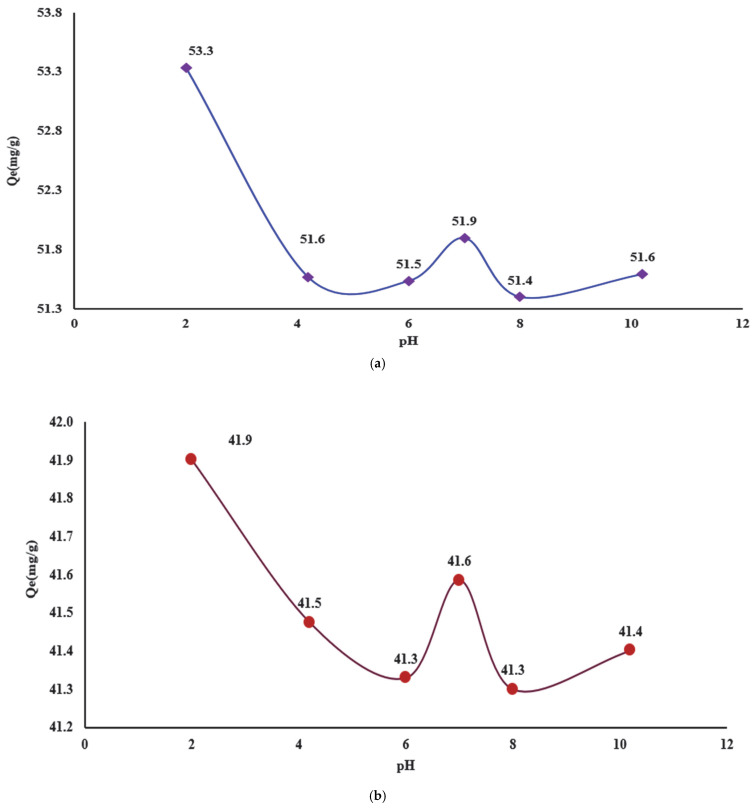
Adsorption of the complexing agent in function of the pH solution on the MS (**a**) and CELL (**b**).

In the experimental studies, initially the retention of the complexing agent (DR 23) on the cellulosic materials was studied based on the pH of the aqueous solution. The results obtained are presented in Figure 4a,b.

The obtained results suggest that the adsorption of the complexing agent on MS and CELL is influenced by the pH of the aqueous matrix. The complexing agent is an anionic compound containing two sulfonic groups that at pH = 2 is dissociated. Thus, an increase in the adsorbed amount is observed at pH = 2 with 53.3 mg/g for MS (Figure 4a) and 41.9 mg/g for CELL (Figure 4b), which decreases with pH increasing at 4 and 6, reaching a new adsorption maximum at pH = 7 (51.9 mg/g for MS and 41.6 mg/g for CELL) and also at pH = 10 (51.6 mg/g MS and 41.4 mg/g for CELL).

This behavior can be attributed to the fact that, in a strongly acidic environment, the hydroxyl groups present in cellulose structure are protonated and became involved in electrostatic interactions with the sulfonic groups (SO_3_^2−^) of the complexing agent DR 23. As the pH of the buffer solutions increases, the deprotonation of the hydroxyl groups occurs until their dissociation, leading to a decrease in the degree of adsorption observed due to the electrostatic repulsions that arrive if we take into account the behavior of the ionizable groups at pH 4 and 5. Also, at pH 7, the obtaining of a new maximum of adsorption can be explained by reporting to the adsorbent mass which, except for the adsorption process specific to the ionizable groups existing in the structure of the tested materials (specific to the hydroxyl groups), has a charge equal to zero, and the adsorption of the DR 23 is achieved by physical interactions or diffusion in the structure of the tested materials.

Thus, we can conclude that the efficiency of the complexation process is governed mainly by electrostatic interactions. Moreover, at pH > 2, the retention of the complexing agent can be achieved through hydrogen bonds as well as through other physical interactions, since new maxima of adsorption were obtained. Based on these observations, it was established that the optimal pH with the most significant adsorption capacities of 53.3 mg/g for MS and 41.9 mg/g for CELL at pH = 2 was achieved. This value was used in the subsequent experimental studies for the complexation of the cellulose materials.

### 3.4. Influence of Contact Time Between the MS/CELL and DR 23

Another parameter studied was the contact time (*t*) necessary to reach the equilibrium state between the liquid and solid phases. The experimental results are presented in Figure 4 and were obtained using the following experimental conditions: at 315 mg DR 23/L, at pH = 2, and contact time varying from 15, 30, 45, 60, 75 to 90 min on 0.05 g MS/CELL.

**Figure 5:**
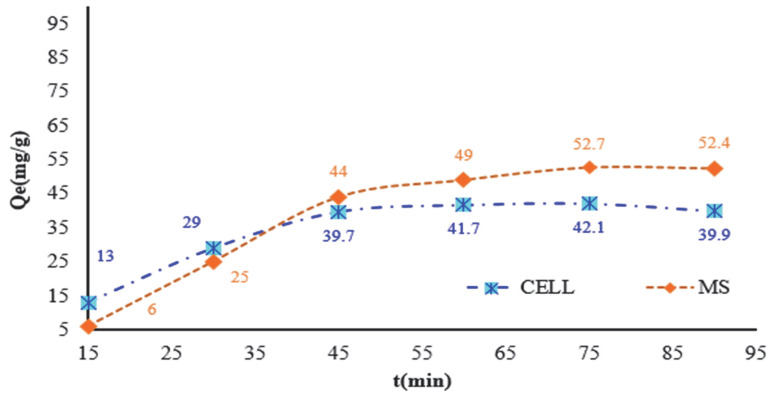
Influence of the contact time required to complex the cellulose existing in the shredded maize stalk and synthetic cellulose.

From Figure 5 it can be observed that the contact time influences the adsorption process of DR 23 on the tested cellulosic materials. Thus, as the contact time increases, the amount of complexing agent (*Q_e_*) increases on the mass of adsorbent material. This increase is more evident in the first stage; accordingly, this initial stage lasts up to 45 min, during which the amount of complexing agent retained is 39.7 mg/g of DR 23 on CELL and 44 mg/g DR 23 on MS. After this stage, the adsorption process of the complexing agent becomes significantly slower, and the adsorption capacity of DR 23 increases up to 52.4 mg/g on MS and 39.9 mg/g for CELL. Based on these experiments it can be concluded that a contact time of 75 min is sufficient to reach the equilibrium state for both cellulosic materials studied.

### 3.5. Influence of the Initial Concentration of the Complexing Agent on the MS and CELL and Stability of Complexing Materials

**Figure 6:**
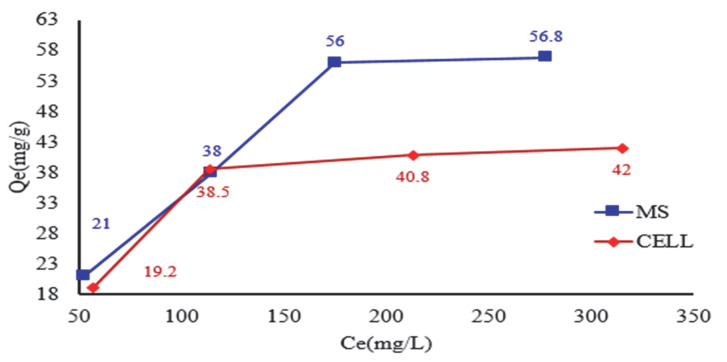
Influence of initial concentration of DR 23 for adsorption of MS and CELL.

The adsorption process of DR 23 on the MS and CELL (Figure 6) was achieved by gradually increasing the initial concentration of DR 23. It was found that the adsorption on the studied celluloses is influenced by the initial concentration varying from 105 to 420 mg/L. The adsorption capacity values in the case of DR 23 adsorption on MS were 21 mg DR 23/g MS, 38 mg DR 23/g MS, 56 mg DR 23/g MS and 56.8 mg DR 23/g MS. For CELL, the experimental values were as follows: 19.2 mg DR 23/g CELL, 38.5 mg DR 23/g CELL, 41 mg DR 23/g CELL and 42 mg DR 23/g CELL.

It is known that when the complexing material exceeds its adsorption capacity, it is considered exhausted and must be regenerated for a new adsorption/desorption step. Furthermore, once the complexing materials are exhausted, the metal ions in the testing solutions are not retained, and this aspect is monitored by measuring their concentrations in the supernatant.

Initially, the stability of the complexing materials was checked in the presence of the desorption agents 2 M HCl and NaOH. The complexing materials were weighed and transferred into an Erlenmeyer flask to which the previously mentioned desorption agents were added and stirred for 30 min. At the end of the stirring time, the materials were filtered and the filtered solutions were analyzed spectrometrically. The amount of complexing agent in the eluent solution was checked based on the following calibration curve: A = 0.0339C + 0.0736.

Stability in acidic and basic environments was chosen because wastewaters are mostly acidic, while at pH > 7 they are basic or alkaline. It was found that the complexing agent DR 23 remains fixed in the mass of the two celluloses when the two desorption agents were added; therefore, they can be regenerated when they are involved in the retention of metal ions using one of the two desorption agents tested.

### 3.6. Testing of Complexing Materials for Metal Ions Adsorption as a Function of pH Medium

**Figure 7:**
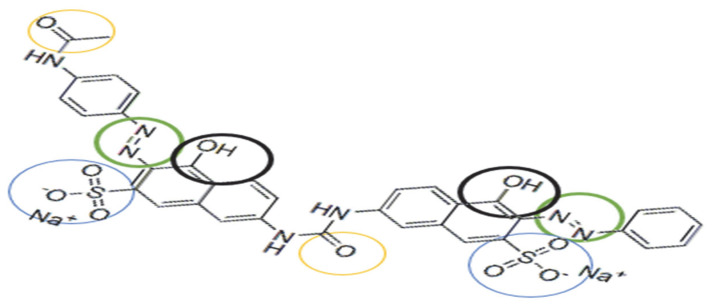
Chemical structure of the complexing agent used for metal ions complexation.

The functional groups present in the structure of the complexing agent (Figure 7) can demonstrate an affinity for metal ions by binding the metal ions through a complexation or chelation mechanism.

Thus, the adsorption mechanism between the metal and the complexing materials can be explained by considering the following hypotheses: (i) DR 23 contains two azo groups (–N=N–) that can bind metals when the azo groups are donating an electron pair, chelating or complexing the metal; (ii) by diffusion in the porous structure of the complexing materials; or (iii) by ion exchange between the –SO_3_^2−^ groups of DR 23 that are not involved in the first adsorption stage of DR 23. Simultaneously, the two materials subjected to complexation contain –OH groups. Also, the MS has in its structure other macro components containing groups such as carboxyl –COO^−^, amino (–NH_2_), sulfhydryl (–SH), etc., which can retain metal ions from the solution through a complexation mechanism [43].

It is known from literature studies that complexing materials change their degree of dissociation in function based on the pH of the solution used [42]. Thus, pH influences not only the solubility of metal ions and organic compounds but also the degree of dissociation of the functional groups present in the structure of the cellulosic material studied in adsorption experiments.

Therefore, to avoid the precipitation of metal ions at higher pH levels > 8, where most metal ions precipitate in the form of hydroxides and the adsorption process is not involved in the adsorption of metal ions, buffer solutions at pH = 2.0; 4.2; 6.0; 8.0 and pH = 10.2 were used.

**Figure 8:**
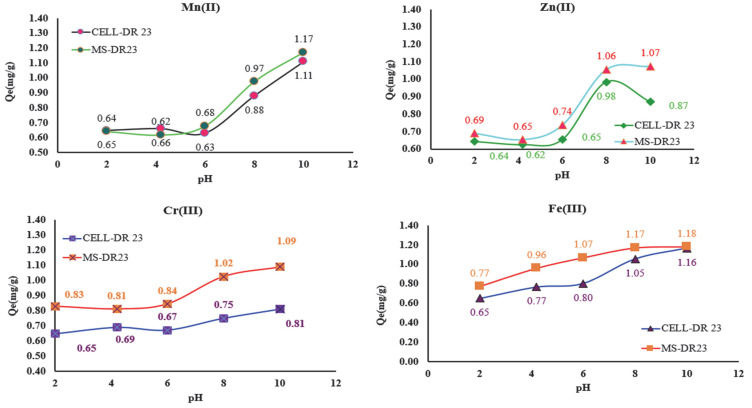
Adsorption behavior of metal ions on complexing materials at different pH values of aqueous matrix.

The new complexing materials (MS-DR 23 and CELL-DR 23) were tested for the removal of metal ions from aqueous matrices as well as to evaluate their applicability in wastewater decontamination experiments at different pH values (Figure 8). Thus, the influence of pH on the adsorption process of metal ions on MS/CELL and in the DR 23 form was studied at pH = 2.0; 4.2; 6.0; 8.0 and 10.2 using 2.5 mg/L M^x+^, 0.05 g of CELL- DR 23 and MS-DR 23 along with 0.03 mL buffer solution.

From the data presented in Figure 8, it can be seen that the retention of Zn^2+^, Mn^2+^, Cr^3+^ and Fe^3+^ on the surface of MS-DR 23 and CELL-DR 23 occurs with the highest efficiency at pH 8 and 10. It is also observed that the best adsorption capacity of MS-DR 23 and CELL-DR 23 was reached at pH = 10. Taking into consideration the *Q_e_*(mg/g) values at pH = 10, the affinity series Fe^3+^ > Mn^2+^ > Cr^3+^ > Zn^2+^ was obtained for MS-DR 23. Meanwhile, for CELL-DR 23, the affinity obtained was Fe^3+^ > Mn^2+^ > Zn^2+^ > Cr^3+^.

### 3.7. Study of Contact Time Between Metal Ions and Complexing Materials

The contact time between the two phases (liquid/solid) is a decisive parameter of the adsorption process that must be studied. The experimental results obtained in the study of the influence of the contact time between MS-DR 23 and CELL-DR 23 and aqueous solutions containing Zn^2+^, Mn^2+^, Cr^3+^ and Fe^3+^ were evaluated, and the results are presented in Figure S1 and Table S1 from the Supplementary Materials.

In this study, it was found that with the increase of initial pH from 2 to 10, of the mixtures subjected to adsorption, the adsorption capacity of the two functionalized materials studied increased. This variation is determined by the fact that with the increase in the pH of the initial solution, the dissociation of the carbonyl (-C=O) and hydroxyl (-OH) groups existing in the structure of the DR 23 complexing agent occurs, which become negatively charged and can bind the positive metal ions (M^x+^) in the solution subjected to adsorption. In order to keep the degree of dissociation of the functional groups relatively constant, a carbonate buffer solution (pH = 10) was used. Taking into account the best *Q_e_* value, all adsorption studies presented subsequently were carried out at pH = 10. The contact time necessary to reach the equilibrium state was studied by batch method in the range of 15 to 90 min. The concentrations of the supernatant solutions obtained at 15, 30, 45, 60, 75 and 90 min, respectively were determined using the calibration line obtained for each metal studied separately in the concentration range of 1–5 mg/L. In this regard, the calibration lines A = 0.2342C + 0.2508 for Zn^2+^, A = 0.156C + 0.055 for Mn^2+^, A = 0.0449C + 0.0354 for Cr^3+^ and A = 0.2342C + 0.2508 for Fe^3+^ were used to determine the concentration of the studied metal ions retained on the mass of complexing materials.

Thus, according to the experimental results, the amount of Zn^2+^, Mn^2+^, Cr^3+^ and Fe^3+^ fixed in the mass of CELL-DR 23 and MS-DR 23 increases with the increasing in contact time and remains constant when equilibrium is reached. It is observed that this increase is more pronounced in the first stage and varies depending on the metal ions studied. In the first stage, the adsorption capacity increased from 0.5 to 1.08 mg/g for Mn^2+^, from 0.6 to 1.2 for Fe^3+^, from 0.24 to 0.9 for Cr^3+^, and from 0.18 to 0.93 for Zn^2+^ during adsorption on CELL-DR 23.

The experimental results for MS-DR 23 in the first stage, varying the contact time and keeping the rest of the operational parameters constant, resulted in *Q_t_* varied as follows: from 0.24 to 1.07 mg/g Mn^2+^, from 0.54 to 1.14 mg/g for Fe^3+^, from 0.36 to 1.2 mg/g for Cr^3+^ and from 0.2 to 1.1 mg/g for Zn^2+^ ions.

After this first stage, the adsorption of metal ions becomes slower, and we observed a flattening trend due to reaching chemical equilibrium. The retained percentages for Zn^2+^, Mn^2+^, Cr^3+^ and Fe^3+^ vary insignificantly for both tested materials, results which are presented in Table S1 from the Supplementary Materials.

Based on these observations, any time greater than 60 min can be used in subsequent adsorption studies, but considering that the structure of functionalized polymers is not sufficiently homogeneous and assuming that access of metal ions to the functionalized groups would be more difficult, a contact time of 90 min would be sufficient to reach the equilibrium state in order to evaluate the adsorption capacity of the tested materials at maximum capacity.

### 3.8. Study of the Initial Metal Ion Concentration on the Binding Capacity of Complexing Materials

Simultaneously, experimental studies at equilibrium were conducted using the batch method. For this purpose, we used 0.05 g of CELL-DR 23 and MS-DR 23 and MS-DR 23 and 0.05 L of 0.5; 1; 1.5; 2; 2.5; 3 and 4 mg/L pH = 10 at 175 rpm for 90 min.

The adsorption capacities (*Q_e_*, mg/g) of metal ions retained on 0.05 g of CELL-DR 23 were 0.25; 0.54; 0.82; 1.02; 1.14; 1.20; 1.21 mg/g for Mn^2+^.

For Fe^3+^ adsorption capacity, they were 0.29; 0.58; 0.88; 1.15; 1.23; 1.20 and 1.20 mg/g, respectively. In the case of Cr^3+^ ions, the adsorption capacities found were 0.2; 0.5; 0.7; 0.8; 0.9; 1.0 and 1.02.

Also, during the investigation of Zn^2+^ ions adsorption on CELL-DR 23, the following values were obtained: 0.25; 0.51; 0.70; 0.87; 0.93; 1.02 and 1.08 mg/g.

At the same time, in the case of MS-DR 23, the following adsorption capacities were calculated for the studied metal ions, obtaining the following values: 0.28; 0.57; 0.85; 1.14; 1.26; 1.26 and 1.32 mg/g for Mn^2+^. For Fe^3+^, *Q_e_* were determined to be 0.30; 0.59; 0.89; 1.18; 1.24; 1.25 and 1.26 mg/g respectively.

In case of Cr^3+^, the adsorption capacity calculated were 0.30; 0.60; 0.80; 1.10; 1.17; 1.2 and 1.22 mg/g. For Zn^2+^, the adsorption capacity varied: 0.26; 0.53; 0.66; 0.86; 0.99; 1.06 to 1.13 mg/g.

**Figure 9:**
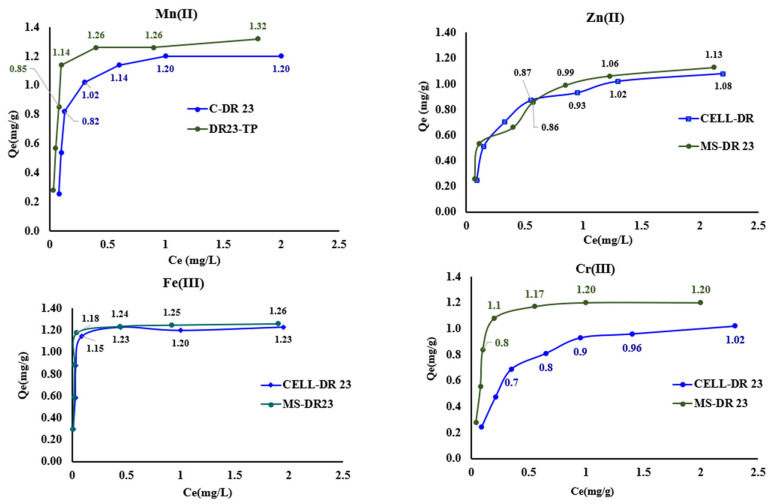
Experimental isotherms for the adsorption of Zn^2+^, Mn^2+^, Cr^3+^ and Fe^3+^ ions for CELL-DR 23 and MS-DR 23, respectively, at pH = 10 of the tested solution.

Analyzing the results presented in Figure 9, it is found that the isotherms obtained experimentally regarding the adsorption of Zn^2+^, Mn^2+^, Cr^3+^ and Fe^3+^ on CELL-DR 23 and MS-DR 23 are nonlinear for the studied concentration range, observing two main adsorption regions.

The first region corresponded to 0.5–2 mg/L, followed by the saturation level at 3 and 4 mg/L for Zn^2+^, Mn^2+^, Cr^3+^ and Fe^3+^.

At the same time, it was found that MS-DR 23 has a better adsorption capacity than the CELL-DR 23, and this increase is all the more evident at higher *C_i_* of Zn^2+^, Mn^2+^, Cr^3+^ and Fe^3^ in the tested solution.

### 3.9. Study of the Langmuir Adsorption Isotherm

The relationship between the amount of metal ion retained per unit mass of adsorbent material and the corresponding concentration of the solute at equilibrium represents the adsorption isotherm.

Thus, the experimental data obtained at equilibrium were modeled based on the Langmuir mathematical model that describes the adsorption process on homogeneous surfaces until a saturation is obtained on the surface of the tested material.

The parameters of the Langmuir isotherm were determined graphically from the slope and intercept of the graphical representation *C_e_/Q_e_* = f(*C_e_*). The results obtained are presented in Figure S1 from Supplementary Materials.

From the data presented in Table 1, it is observed that the values of the R^2^ are greater than 0.9800. This indicates that the CELL-DR 23 and MS-DR 23, respectively, are considered adsorbents with a homogeneous surface on which Zn^2+^, Mn^2+^, Cr^3+^ and Fe^3+^ can be retained through a specific complexation and adsorption interactions until obtaining a monolayer that covers the surface of CELL-DR 23 and MS-DR 23.

Based on the Langmuir model, the maximum adsorption capacities *Q_max_* (mg/g) were determined, obtaining the following values: 1.31 mg/g Mn^2+^, 1.27 mg/g Fe^3+^, 1.15 mg/g Cr^3+^ and 1.21 mg/g Zn^2+^ for the CELL-DR 23. For MS-DR 23, the *Q_max_* values determined by fitting the experimental data were 1.36 mg/g Mn^2+^, 1.27 mg/g Fe^3+^, 1.28 mg/g Cr^3+^ and 1.29 mg/g Zn^2+^. It is noted that the *Q_max_* (mg/g) values of MS-DR 23 are higher than for CELL-DR 23, results presented in Table 1.

One possible explanation can be that the MS-DR 23 was better loaded with complexing agent, and also, chemical groups from its structure that were not employed in DR 23 adsorption can be employed to complexing metal ions. Moreover, the value of the R_L_ constant (g/L) between 0 and 1 indicates a favorable adsorption process of the studied metal ions on MS-DR 23 and CELL-DR 23.

### 3.10. Study of Regeneration of Exhausted Complexing Materials with Metal Ions

The desorption eluent has an important role in the regeneration process of the exhausted material as well as for the recovery of useful constituents. It provides the breakage of the metal from the complexing material. Thus, the eluent concentration for desorption should be chosen following experimental studies because a high concentration may affect the structure of the complexing material and a low concentration may have a slow desorption requiring a large volume of eluent [44].

**Figure 10:**
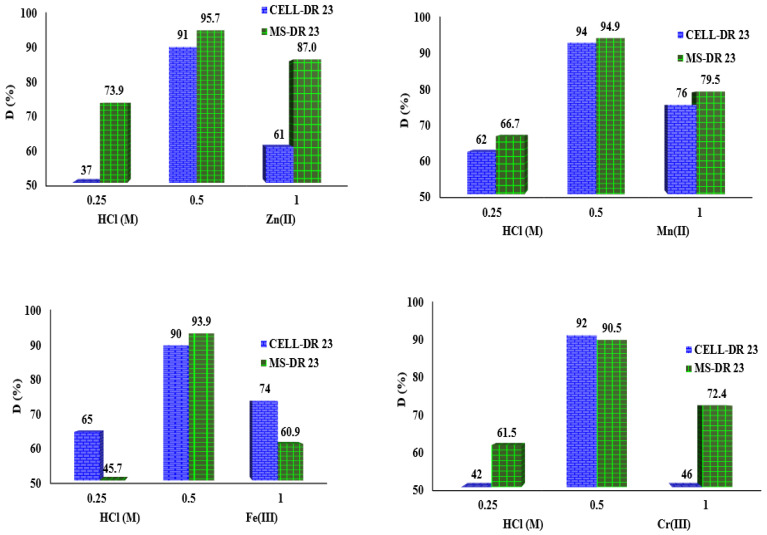
Desorption of Mn^2+^, Fe^3+^, Cr^3+^ and Zn^2+^ from MS-DR 23 and CELL-DR 23 using 0.25, 0.5 and 1 M HCl.

In this part, the exhausted materials were regenerated with 0.25, 0.5 and 1 M HCl to release Mn^2+^, Fe^3+^, Cr^3+^ and Zn^2+^ loaded on MS-DR 23 and CELL-DR 23. Herein, the MS-DR 23 and CELL-DR 23 were subjected to adsorption/recovered/regenerated/reused in a new adsorption/desorption process. The complexing materials were subjected to further loading by the batch method with 2 mg/L of Mn^2+^, Fe^3+^, Cr^3+^ and Zn^2+^, respectively. After 90 min of stirring at 175 rpm (T = 25 ± 2 °C) in batch mode, the mixtures were centrifuged, filtered and solid phase was kept for desorption studies. For the MS-DR 23, the tested samples used were 0.05 g of 1.17 mg/g Mn^2+^, 1.2 mg/g Fe^3+^, 1.09 mg/g Cr^3+^ and 1.07 mg/g Zn^2+^, respectively. For the reuse of CELL-DR 23, the solid samples employed were loaded with 1.11 mg/g Mn^2+^, 1.16 mg/g Fe^3+^, 0.81 mg/g Cr^3+^ and 0.87 mg/g Zn^2+^ and tested. Following the desorption studies, it is observed that when 0.5 M HCl is used, Mn^2+^, Fe^3+^, Cr^3+^ and Zn^2+^ existing on the MS-DR 23 and CELL-DR 23 are desorbed in a proportion over 90%. The data obtained at desorption of Mn^2+^, Zn^2+^, Fe^3+^ and Cr^3+^ are presented in Figure 10.

For the CELL-DR 23 loaded with Mn^2+^, Fe^3+^, Cr^3+^ and Zn^2+^, the effective desorption percentages were detected as follows: 91% Zn^2+^, 94% Mn^2+^, 90% Fe^3+^ and 92% Cr^3+^ when 0.5 M is used. Simultaneously, a decrease in the desorption efficiency was observed when 0.25 and 1 M HCl was used for both tested complexing materials.

Considering the results obtained, the following regeneration studies for materials regeneration loaded with metals (MS-DR 23-M^x+^ and CELL-DR 23-M^x+^) were performed using 0.5 M HCl.

### 3.11. Reutilization of Complexing Materials in Multiple Adsorption/Desorption Cycles

**Figure 11:**
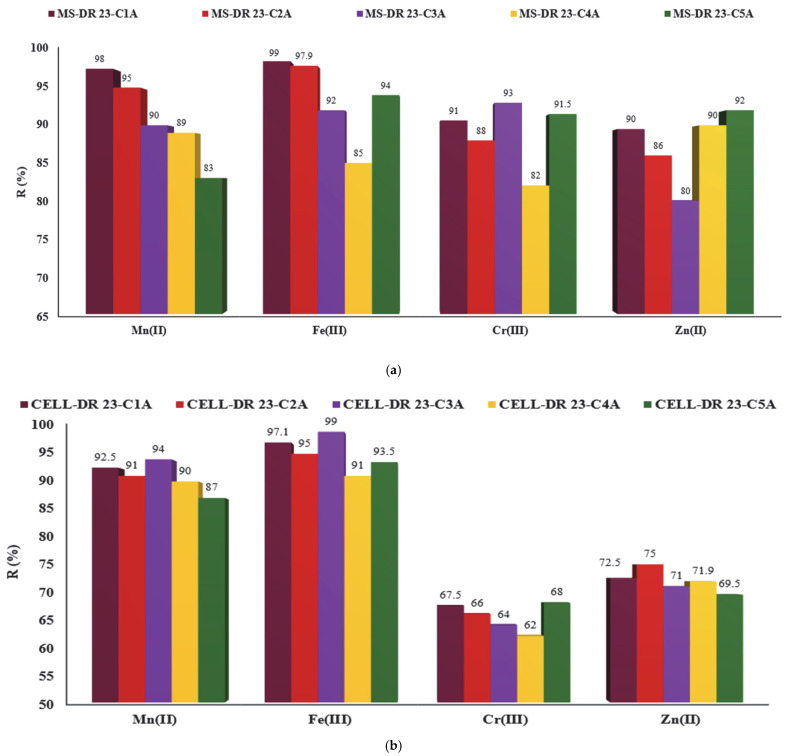
Reutilization of MS (**a**) and CELL (**b**) in DR 23 form in five adsorption cycles.

Acid effluents polluted with metal ions can be treated by applying the principles of circular economy and involve the reducing of metal concentration and recovering the materials for subsequent use. For this propose, CELL-DR 23 and MS-DR 23 were stirred with 2 mg/L mixed solution of Mn^2+^, Fe^3+^, Cr^3+^ and Zn^2^, and from the supernatant solutions, the concentration of metal ions was determined by AAS method while the solid phases were subjected to regeneration with 0.5 M HCl solution in order to recover and reuse them in a new adsorption/desorption cycle. The results obtained are presented in Figure 11a,b.

So, the chelated materials (CELL-DR 23 and MS-DR 23) loaded with Mn^2+^, Fe^3+^, Cr^3+^ and Zn^2+^ were regenerated and reused in five adsorption/desorption cycles. It was found that for a new reuse from one to five adsorption cycles, a variation was obtained more evident for MS-DR 23 if we compare with CELL-DR 23, as is presented in Figure 11a,b.

### 3.12. Applications of Complexing Materials on Tannery Wastewater Matrix

During this study, the best adsorption capacity of the complexing materials was found at pH = 10, and the applications of complexing materials on tannery wastewater matrix were performed at this value.

Considering this, when adsorption studies were conducted on tannery wastewater, a mixture in a 1:1 ratio with the acetate buffer solution (pH = 10) was created, obtaining a pH = 9.9 of the mixed solution. At the same time, *C_i_* of metal ions before adsorption on MS-DR 23 and CELL-DR 23 was determined, obtaining 1.9 ± 1.75 mg/L Cr^3+^, 1.2 ± 1.06 Fe^3+^, 0.7 ± 0.55 mg/L Mn^2+^ and 0.35 ± 0.22 mg/L Zn^2+^, respectively.

It is also known that the adsorption capacity of complexing materials decreases for metals with a large ionic radius due to the steric effect. Thus, metals with a small ionic radius enter in the porous structure of the complexing materials and interact with the functional groups present in their structure. For metal ions studied, ionic radius decreased as follows: 0.55 Å Fe^3+^ < 0.62 Å Cr^3+^ < 0.67 Å Mn^2+^ < 0.74 Å Zn^2+^. Therefore, the affinity sequence of the studied metals could be governed by their ionic radius.

**Figure 12:**
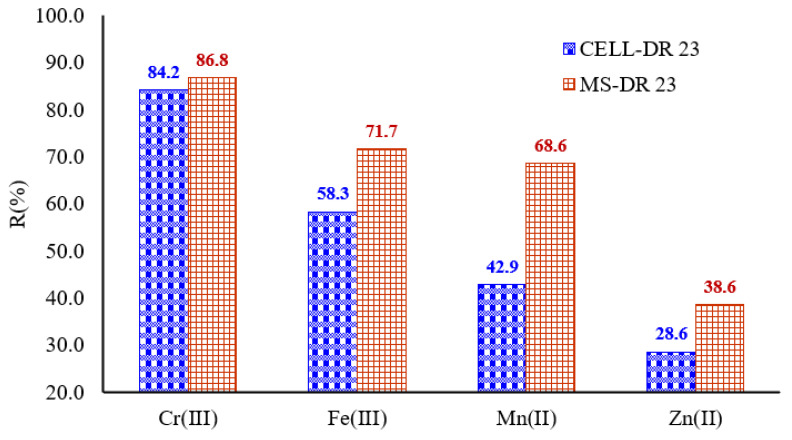
Adsorption of metals from tannery wastewater on CELL-DR 23 and MS-DR 23, respectively.

For metals that possess the same charge, the tendency is to retain them according to their ionic radius. This means that for divalent metal ions, the metal with the smallest ionic radius should easily diffuse into the structure of the complexing cellulose polymers and be involved in the complexation reaction with the functional groups present in the structure of chemically modified materials. Analyzing the results presented in the Figure 12, the CEEL-DR 23 and MS-DR 23 are promising materials for the simultaneous removal of the Mn^2+^, Fe^3+^, Cr^3+^ and Zn^2+^, one in the presence of the other.

Additionally, the removal percentage exceeded 28% for the studied metals; these results are influenced by the *C_i_* of Mn^2+^, Fe^3+^, Cr^3+^ and Zn^2+^ present in the wastewater matrix.

So, it was observed that MS-DR 23 and CELL-DR 23 significantly reduced the concentration of metals as follows: 68.6% Mn^2+^, 71.7% Fe^3+^, 86.8% Cr^3+^ and 38.6% Zn^2+^ for MS-DR 23. Simultaneously, CELL-DR 23 indicated a rate of adsorption by 42.9% Mn^2+^, 58.3% Fe^3+^, 84.2% Cr^3+^ and 28.6% Zn^2+^. Taking into consideration those results, we can see that the concentration gradient of metals influences the adsorption process on developed materials.

## 4. Conclusions

In conclusion, as observed, one of the most significant influences on the adsorption process regarding the retention of the complexing agent DR 23 on the two celluloses is determined by the pH of the aqueous solution, which impacts the hydroxyl groups as well as the ionization process of the DR 23 complexing agent. As observed at pH = 2, the retention of the complexing agent occurs with the highest efficiency.

Thus, pH affects the adsorption process of the complexing agent by influencing the degree of dissociation of the functional groups of the surface-binding centers in the structure of the adsorbent celluloses, leading to the modification in the equilibrium characteristics of the adsorption process.

Thus, at sufficiently low pH values, the surface of cellulosic materials is protonated and the DR 23 molecules are negatively charged, which attracts them to the cellulose-binding centers. Due to electrostatic attraction forces, the DR 23 molecule is adsorbed on the cellulose mass.

Also, analyzing the results obtained by varying each experimental parameter evaluated in the adsorption of Zn^2+^, Mn^2+^, Cr^3+^ and Fe^3+^ ions on CELL-DR 23 and MS-DR23, respectively, it can be said that the adsorption capacity increases with pH increasing in the studied solution; the increase in the initial concentration of Zn^2+^, Mn^2+^, Cr^3+^ and Fe^3+^ ions in the concentration range 0.5–4 mg/L leads to an increase in the adsorption capacity of CELL-DR 23 and MS-DR23, respectively; and the increase in contact time between the phases of the adsorption system leads to an increase in the amount of metal ion on both CELL-DR 23 and MS-DR 23. This increase is more pronounced in the first adsorption stage, which is observed up to 60 min; after this stage, the adsorption process becomes slow and the adsorption capacity varies insignificantly, indicating that equilibrium has been reached.

Cellulosic complexing materials show a good stability in acidic and basic environments up to the tested concentration of 2 M. Regeneration studies were conducted with increased efficiency when using 0.5 M HCl. At the same time, the complexing materials have proven their susceptibility to multiple reuses. In conclusion, for equal concentrations of metal ions, the complexing polymers exhibit a preferential affinity for Cr^3+^ and Fe^3+^ in the presence of the two divalent metals (Mn^2+^ and Zn^2+^). Moreover, the concentration gradient influenced the adsorption capacity of the metal ions evaluated on the tested materials.

So, this study is dedicated to loading and complexing cellulose materials and application of complexing celluloses to metal removal from aqueous matrices.

## Figures and Tables

**Table 1:** Parameter of Langmuir model obtained for adsorption of Mn^2+^, Zn^2+^, Fe^3+^ and Cr^3+^ using complex cellulose materials.

Model	Mn^2+^	Fe^3+^	Cr^3+^	Zn^2+^	Mn^2+^	Fe^3+^	Cr^3+^	Zn^2+^
Langmuir	CELL-DR 23				MS-DR 23			
R^2^	0.9825	0.9999	0.9978	0.9953	0.9978	0.9999	0.9968	0.9922
*Q_max_* (mg/g)	1.31	1.27	1.15	1.21	1.36	1.27	1.28	1.29
R_L_ (g/L)	0.07	0.005	0.14	0.12	0.03	0.005	0.04	0.13

## Data Availability

Data are available upon request from the authors.

## Supplementary Materials

The following supporting information can be downloaded at: https://www.mdpi.com/article/10.3390/polym17111467/s1, Figure S1: Influence of contact time for metal ions removal onto MS-DR 23 and CELL-DR 23; Table S1: Percentage removal of metal ions onto MS-DR 23 and CELL-DR 23.
