# Supplementary material for: Green Chemistry Applications Using Complexing Materials for Water Treatment
Source: Polymers (Basel). 2025 May 25;17(11):1467. doi: 10.3390/polym17111467 (PMC12157184; doi:10.3390/polym17111467)
Supplement: Supplementary file 1 [file polymers-17-01467-s001.zip › polymers-3634889-supplementary.pdf]

polymers

Supplementary Material

Green Chemistry Applications Using Complexing Materials for Water Treatment

Nicoleta Mirela Marin [1,2,3]

[1] National Research and Development Institute for Industrial Ecology ECOIND, Street Podu Dambovitei no. 57-73, District 6, 060652 Bucharest, Romania
[2] Department of Oxide Materials Science and Engineering, National University of Science and Technology Politehnica Bucharest, 1–7 Gh. Polizu, 060042 Bucharest, Romania;
[3] Department of Analytical and Physical Chemistry, University of Bucharest, 4-12 Regina Elisabeta Bd., 030018 Bucharest, Romania
* Correspondence: tomagalaon@yahoo.com (TG), luoanapascu@yahoo.com (L.F.P.)

Figure S1. Influence of contact time for metal ions removal onto MS-DR 23 and CELL-DR 23.

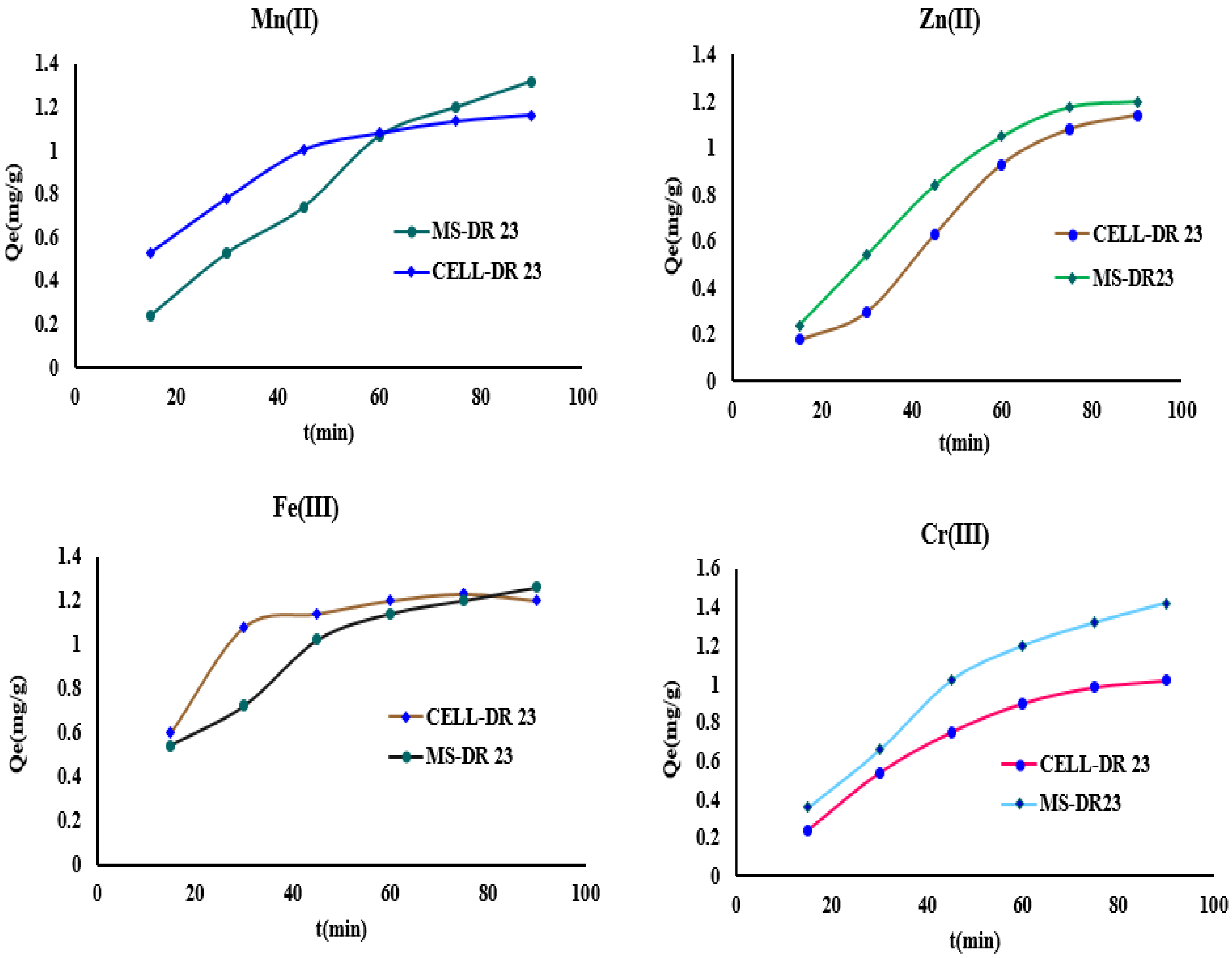

 https://doi.org/10.3390/polym17111467

Table S1. Percentage removal of metal ions onto MS-DR 23 and CELL-DR 23.

Time(min)	Mn(II)	Fe(III)	Cr(III)	Zn(II)	Mn(II)	Fe(III)	Cr(III)	Zn(II)
	R(%) CELL-DR 23				R(%) MS-DR 23			
15	22	25	10	8	10	22.5	15	10
30	33	45	23	13	22	30	27.5	22.5
45	42	48	31	26	30.8	42.5	42.5	35
60	45	50	38	39	44.5	47.5	50	43.8
75	47	51	41	45	50	50	55	49
90	49	50	43	48	55	52.5	59.3	50
